# The impact of job insecurity on long-term self-rated health – results from the prospective population-based MONICA/KORA study

**DOI:** 10.1186/s12889-018-5621-4

**Published:** 2018-06-18

**Authors:** Amira Barrech, Jens Baumert, Harald Gündel, Karl-Heinz Ladwig

**Affiliations:** 1 Department of Psychosomatic Medicine and Psychotherapy, Universitätsklinik Ulm, Ulm, Germany; 2 Institute of Epidemiology II, Helmholtz Zentrum München, German Research, Center for Environmental Health, Ingolstädter Landstr. 1, 85764 Neuherberg, Germany; 3 Department of Psychosomatic Medicine and Psychotherapy, Klinikum rechts der Isar, Technische Universität München, Munich, Germany

**Keywords:** Job insecurity, SRH, Work-related stressors, Successful aging, Working population, Epidemiology

## Abstract

**Background:**

Job insecurity has been associated with impaired self-rated health (SRH) in cross-sectional studies, but prospective findings with short, medium and long-term follow-up yielded mixed findings. Therefore, the aim of the present study was to assess the long-term association between perceived job insecurity and SRH, after controlling for baseline levels of health status and life-style choices. Furthermore, three different follow-up periods (14, 19 and 24 years) were considered.

**Methods:**

Data were derived from the prospective population-based MONICA/KORA cohort study (southern Germany). *N* = 4356 participants (2622 men and 1734 women), aged between 25 and 64 years at baseline, were included in the sample, mean follow-up was after 19.1 years. Job insecurity, SRH and risk factors were assessed at baseline during three independent surveys (1984–1995). SRH was additionally assessed in 2009. The association of job insecurity and impaired SRH at follow-up was estimated using logistic regression analyses.

**Results:**

Overall, perceiving job insecurity at baseline was significantly associated with a 20% higher risk of developing impaired SRH at follow-up in the pooled analysis (OR = 1.20, 95% CI = 1.01–1.43, *p* = .034), even after controlling for baseline SRH, socio-demographic characteristics, lifestyle, clinical and work-related factors. The association was strongest and significant after 14 years (OR = 1.58, 95% CI 1.17–2.13, *p* = .003) and weaker and not significant to 19 (OR = 1.20, 95% CI 0.89–1.62, *p* = .24) and 24 years (OR = 0.98, 95% CI 0.73–1.32, *p* = .89) of follow-up in the fully adjusted models.

**Conclusions:**

We found that perceived job insecurity during working life was independently and significantly associated with impaired SRH both cross-sectionally as well as after 14 years, but not after 19 and 24 years.

**Electronic supplementary material:**

The online version of this article (10.1186/s12889-018-5621-4) contains supplementary material, which is available to authorized users.

## Background

The past decades have seen economic crises, rising unemployment and a global shift away from stable employment patterns to non-standard forms of work on the macro-level [1]. Reflecting the changing economic environment, organizational change and reorganizations have become the norm in working environments [2]. A stable proportion of 16% of employees in the European Union currently report job insecurity [3]. Growing use of flexible forms of employment is expected to lead to an increase [1].

Job insecurity, described as uncertainty concerning the future of an existing employment situation, can be distinguished as either “objective” or “subjective/perceived” job insecurity [4, 5]. Objective job insecurity is assumed in situations that objectively endanger an employee’s job (without reference to individual perceptions), such as the announcement of major lay-offs in a company [4]. Perceived job insecurity on the other hand results from an individual’s subjective appraisal of a given situation (e.g. rumours of lay-offs) and can occur in the absence of an objective trigger [4]. Individuals are more likely to develop job insecurity if the anticipated change is perceived as involuntary and outside their realm of control [4]. To this end, different individuals can come to different conclusions in the same situation [4, 6]. Therefore, the focus of the present study lies on ‘perceived job insecurity’, henceforth referred to as job insecurity. Research to date on antecedents has found that job insecurity is for example associated with younger age, temporary employment and lower socio-economic status [7].

To date, job insecurity has been linked to a number of health outcomes such as for example coronary heart disease [8], depression [9], impaired subjective well-being [10, 11] and poorer self-rated health [11]. Different theoretical frameworks are used to explain the negative effect of job insecurity on health [11], such as Jahoda’s latent deprivation model [12] which posits that job insecurity threatens the satisfaction of important needs (e.g. income, social status) which in turn affects well-being. Likewise, the stressful nature of job insecurity and its health related outcomes are frequently explained along the lines of Lazarus & Folkman’s (1984) transactional stress-model [11, 13]. Hereby, job insecurity is the result of an individual’s (primary) appraisal of a certain situation (e.g. rumours on lay-offs) as being a threat. If it concludes that the consequences resulting from the anticipated change exceed its coping resources (e.g. poor employability), strain is a likely outcome. While individuals who have been informed of their imminent layoff can take active measures that help to cope with the prospect of actual job loss (e.g. by looking for a new job), the uncertainty inherent in job insecurity makes it difficult to prepare for the future [11]. Indeed, job insecurity has been associated with stress-related physiological markers such as an increase in blood-pressure and BMI [11] as well as insulin resistance and higher cortisol concentrations [14].

Self-rated health (SRH), a single question that measures subjective health status, has repeatedly shown to be of a high predictive value for both morbidity and mortality, independent of objective measures of health [15]. Surprisingly however, the exact framing or wording of the question seems to be irrelevant [15]. The high predictive value of self-ratings of health is partly ascribed to the fact that individuals consider an elaborate set of health-relevant information (e.g. diagnosed diseases, health-behaviour) when answering this question, more than can be included in a questionnaire [16]. Moreover, evidence suggests that unspecific afferent bodily sensations (e.g. tiredness, unspecific pain) are interpreted as health indicators and included into the assessment of self-rated health, potentially reflecting subclinical inflammatory activity in the body prior to any clinical diagnosis [15, 16].

Job insecurity, a work-related stressor, has been prospectively associated with impaired SRH in a number of studies [11]. However, some research gaps remain: first, there is conflicting evidence on the quality of the relationship between job insecurity and SRH. A number of cross-sectional population-based, as well as cohort studies from the working environment, have established a concise and stable relationship between job insecurity and impaired SRH [17–22]. A number of prospective studies have further examined this association, predominantly using brief follow-up periods, but findings were mixed. While five studies have evaluated the relationship in the short –term (6 months, 1 year, 2 years, 2,5 years and 3 years) [23–28], only two have considered mid- (5 and 6 years) [28, 29] and long-term (9 and 12 years) [23, 30] periods, respectively. Results from a German sample for example report a significant association between job insecurity and impaired SRH after one as well as three years [26], while in a Danish sample job insecurity was only significantly associated with impaired SRH among women after five years [29]. Interestingly, in studies with long-term follow-up periods significant associations between job insecurity and impaired SRH were only found among participants who were repeatedly exposed to job insecurity [23, 30]. Moreover, the prospective studies only controlled for a very limited number of variables related to health status (e.g. smoking, illness) at baseline [25, 28–30]. Furthermore, in their review on the longitudinal health effects of job insecurity, De Witte et al. call for future research that “…should try to find out whether effects appear or disappear after specific time lags or not, and should more systematically tests various time lags..”.

Therefore, the aim of the present study was to extend the knowledge base in two ways: first, to assess the long-term association between job insecurity and impaired SRH after controlling for a number of relevant covariates, including baseline levels of health status and life-style choices. Second, to test the long-term association between job insecurity and impaired SRH across three different follow-up periods (14, 19 and 24 years). The anticipated increase in flexible forms of employment as well as a longer working life (due to increased life expectancy) call for a better understanding of the long-term association of job insecurity with impaired SRH, a valuable proxy for objective health due to its high predictive value and ease of administration.

## Methods

### Setting and sample description

The present study was based on the population-based MONICA/KORA (Monitoring of Trends and Determinants in Cardiovascular Disease/ Cooperative Health Research in the Region of Augsburg) cohort study, which was conducted in the region of Augsburg (Southern Germany) between 1984 and 2009 [31, 32]. The study was designed to have three independent cross-sectional studies (S1-S3) in five-year intervals, enabling analyses of potential trends in cardiovascular risk factors and diseases. For the present study, baseline data was derived from these three independent surveys: 1984/85 (S1), 1989/90 (S2) and 1994/95 (S3) [31]. Their health status was assessed in terms of a variety of diseases (e.g. CVD, diabetes) and conditions at baseline, among them self-rated health [32]. Among them, 11,287 participants were then followed up through a self-reported postal questionnaire within the KORA framework in 2008/2009 (i.e. *n* = 2139 participants could not be contacted and had missing information on follow-up). This lead to a different time-to-follow-up for participants of S1, S2 and S3, respectively. The total cohort consists of 13,426 participants aged 25–74 years at baseline [32].

**Fig. 1:**
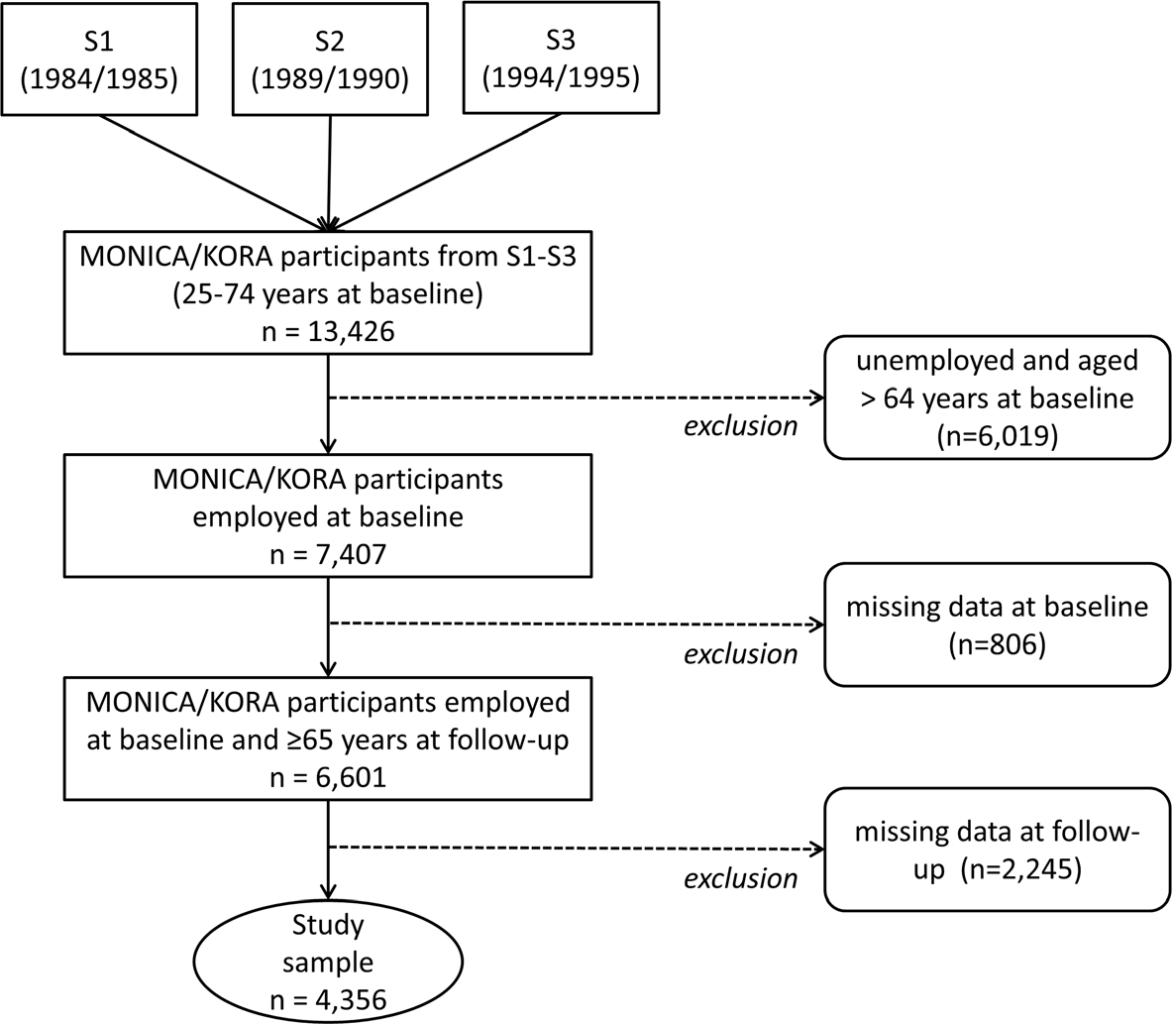
Flow chart of participants

The present study was restricted to participants who were employed and aged 25–64 years at baseline (*n* = 7407). From this subsample, participants with missing information on job insecurity, SRH and any of the risk factors at baseline (*n* = 806) as well as additionally with missing information on self-rated health at follow-up were excluded (*n* = 2245), leading to a final study sample of 4356 participants (2622 men and 1734 women; Fig. 1). Of the 4356 participants, 1336 had participated in S1, 1440 in S2 and 1580 in S3. A drop-out analysis showed that missing information was mainly associated with older age and being male.

Mean age at baseline was 42.4 years (standard deviation (SD) 10.2), ranging from 25 to 64 years; mean age at follow-up was 61.6 years (SD 10.9), ranging from 39 to 88 years. Overall, participants were followed-up after 19.1 years (SD 4.1). The average time-to follow-up for S1 was 24.0 years (SD 0.1), 19.0 years (SD 0.1) for S2 and 14.0 years (SD 0.1) for S3.

### Measures

#### Job insecurity

‘Job insecurity’ was assessed at each baseline assessment (S1-S3 between 1984 and 1995) using a global single item in a self-administered questionnaire (“Do you sometimes worry about whether you will be able to keep your current work position?”), with three answer categories (1 = “yes, frequently”, 2 = “yes, sometimes” and 3 = “no, never”). Participants answering “yes, frequently” or “yes, sometimes” were defined as experiencing job insecurity. In addition, the analyses were repeated using all three reply categories as a sensitivity analysis (Additional file 1: Table S1a).

### Self-rated health

SRH was determined by a face-to-face interview at baseline and by a self-administered questionnaire, or in case of non-response, by a telephone interview at follow-up. SRH was assessed with the question “How would you assess your current physical condition?” with four answer categories (‘very good’, ‘good’, ‘suboptimal’, ‘poor’). Participants answering ‘suboptimal’ or ‘poor’ were defined as reporting impaired SRH.

### Baseline risk factor assessment

Multivariate analyses were adjusted for baseline levels of a set of variables that have previously been associated with either job insecurity (age [7], low educational level [7], physical labour [7]) and/or impaired SRH (age [33], gender [34], low educational level [15], life-style choices [35], obesity [35], chronic cardiometabolic diseases [36], overtime [37], shift work [38], night shifts [38]). Data were collected at baseline by means of questionnaires, standardized face-to-face interviews as well as medical examination, details hereof are provided elsewhere [31, 32].

**Socio-demographic variables:** In addition to gender and age, ‘Low educational level‘ was defined as having less than 12 years of schooling. *Life-style- related variables:* ‘Smoking’ was classified as reporting smoking regularly and at least one cigarette per day on average. ‘Alcohol consumption’ was defined according to the average daily alcohol intake leading to the categories ‘no’ (0 g/day), ‘moderate’ (< 40 g/day for men and < 20 g/day for women) and ‘high’ (≥ 40 g/day for men and 20 g/day for women) based on previous studies regarding cardiovascular and all-cause mortality on recommendations of the WHO [39]. Participants reporting to not engage in sports for at least 1 h per week on a regular basis were categorised as ‘physically inactive’ [40]. Having a body mass index ≥ 30 kg/m^2^ constituted ‘Obesity’ [41]. *Health-related variables:* ‘Chronic cardiometabolic diseases’ were defined as having a history of diabetes, myocardial infarction, stroke or hypertension; no history of any of these diseases meant having no chronic cardiometabolic diseases. These diseases were chosen because of their high prevalence, the validity of their self-reports and their effect on daily life (e.g. medication, diet, frequent doctor visits etc.). *Work-related risk factors* to health: Participants were asked to specify whether their job encompassed overtime, shift work, night shifts and physical labour. Answering ‘often’ or ‘sometimes’ were considered as ‘yes’ and answering ‘never’ as ‘no’.

### Statistical analysis

All analyses were weighted using an inverse probability weighting (IPW) approach [42] stratified for age group (10-year age groups), gender and survey to deal with missing information in the subsample (i.e. all employed participants at baseline between the ages of 25–64 years, *n* = 7407). By this weighting, the analyses are based on the age, gender and survey distribution of the subsample reducing potential bias of missing information which was mainly affected by age and gender. Robust variance estimations appropriate to the weighting scheme were computed using the SAS procedures SURVEYMEANS, SURVEYFREQ, SURVEYREG and SURVEYLOGISTIC.

For unadjusted, descriptive analyses, mean differences of continuous variables in groups were assessed by conducting the *t* test or *F* test. Associations between categorized variables were assessed by using the χ^2^ test.

For adjusted, multiple analyses, logistic regression models were estimated to assess the association of job insecurity with impaired SRH at follow-up. In order to account for the potential influences of health selection on the association between job insecurity and SRH, we control for SRH at baseline as well as a number of health-related covariates. Cumulative additional adjustments were made for age, gender, educational level, survey and self-rated health at baseline (model 1), life-style factors (smoking, alcohol consumption, obesity and physical inactivity; model 2), chronic cardiometabolic diseases (history of diabetes, myocardial infarction, stroke or hypertension; model 3) and work-related factors (overtime, shift work, night work, physical labour; model 4). Furthermore, the logistic regression was stratified by survey in order to assess differences in the association between job insecurity and impaired SRH based on time-to-follow-up. The variable survey can be regarded as indicator for time point of baseline examination or time of follow-up. A *p* value for this potential modification of association was estimated by including interaction terms of job insecurity and survey in model 4 (interaction analysis). Additionally, as sensitivity analyses, the logistic regression was repeated using the original reply categories for job insecurity (three categories).

The c-statistic was used to assess the model fit of the logistic regression models. For all statistical analyses, a p value < 0.05 was considered to be statistically significant. SAS Version 9.2 for Windows (SAS Institute, Cary, NC) was used for all statistical analyses.

## Results

### Descriptive analyses

Among the 4356 included participants (38.6% female) of the present study, 41.8% reported job insecurity at baseline which was higher in men than in women (43.4% versus 36.2%, *p* = .006). Participants reporting job insecurity were slightly younger overall (41.8 vs. 42.9, *p* = .002). Job insecurity was reported more often in participants aged 15–54 years (43.3–47.2% in men and 38.3–44.1% in women) as compared to the oldest age bracket of 55–64 years (30.2% in men and 25.7% in women); overall *p* values were < 0.001 in both genders (data not shown).

At baseline, job insecurity was further significantly associated with having a lower educational level (66.8% vs. 61.7%), working overtime (79.3% vs. 74.9), performing physical labour (43.7% vs. 36.3%) and impaired SRH (19.0 vs. 13.9) (Table 1).

**Table 1:** Baseline descriptive characteristics of the sample (*n* = 4356)*

	Total sample	Job insecurity		
Characteristic	n = 4356 % / mean (SD)	No *n* = 2508 % / mean (SD)	Yes *n* = 1848 % / mean (SD)	*p* value
Age	42.4 (10.2)	42.9 (10.7)	41.8 (9.4)	.002
Female gender	38.6	40.4	36.2	.006
Low educational level	63.9	61.7	66.8	.001
Smoking	28.4	28.9	27.7	.410
Alcohol consumption				.106
No	21.9	22.6	21.0	
Moderate	49.0	47.6	50.9	
High	29.1	29.8	28.1	
Obesity	13.6	13.0	14.6	.150
Physical inactivity	49.7	48.8	51.0	.163
Chronic disease**	29.6	29.7	29.4	.865
Overtime	76.7	74.9	79.3	.001
Shift work	10.5	9.8	11.4	.086
Night work	8.4	8.1	8.9	.381
Physical labour	39.5	36.6	43.7	<.001
Impaired SRH	16.0	13.9	19.0	<.001

*Means (SD) and % were weighted

**History of diabetes, myocardial infarction, stroke or hypertension

### Association of job insecurity with impaired SRH – Main analyses

Table 2 shows the association of job insecurity at baseline with impaired SRH at follow up, adjusted for a number of risk factors. In Model 1 (adjusted for baseline values of age, gender, educational level, SRH and survey), job insecurity was significantly associated with impaired SRH at follow-up (OR 1.20, 95% CI 1.02–1.42), compared to an employment considered as secure. This association was robust to cumulative additional adjustments for life-style factors (model 2) and chronic cardiometabolic diseases (model 3; OR 1.22, 95% CI 1.03–1.44). After additionally controlling for work-related variables (model 4), the OR decreased slightly, while still remaining significant (OR 1.20, 95% CI 1.01–1.43). The model fit assessed by the c-statistic increased between Models 1 and 4, suggesting a slight improvement after inclusion of further covariates and displayed sufficient values overall.

**Table 2:** Association of job insecurity with impaired SRH at follow-up (n = 4356)

Risk factor	Model 1 OR (95% CI)	Model 2 OR (95% CI)	Model 3 OR (95% CI)	Model 4 OR (95% CI)
Job Insecurity	1.20 (1.02–1.42)*	1.22 (1.03–1.44)*	1.22 (1.03–1.44)*	1.20 (1.01–1.43)*
*Socio-demographic characteristics*				
Age (years)	1.04 (1.03–1.05)***	1.04 (1.03–1.05)***	1.04 (1.03–1.05)***	1.04 (1.02–1.05)***
Male gender	0.97 (0.81–1.15)	1.02 (0.85–1.22)	1.05 (0.87–1.26)	1.08 (0.89–1.30)
Low educational level	1.54 (1.28–1.86)***	1.42 (1.18–1.72)***	1.42 (1.17–1.72)***	1.34 (1.10–1.64)**
*Lifestyle characteristics*				
Smoking		1.67 (1.38–2.01)***	1.67 (1.38–2.01)***	1.66 (1.38–2.01)***
Moderate alcohol consumption^a^		0.95 (0.76–1.19)	0.96 (0.77–1.20)	0.97 (0.77–1.21)
High alcohol consumption^a^		1.03 (0.81–1.31)	1.03 (0.81–1.31)	1.02 (0.80–1.30)
Obesity		1.78 (1.42–2.23)***	1.70 (1.36–2.14)***	1.66 (1.32–2.09)***
Physical inactivity		1.23 (1.04–1.46)*	1.23 (1.04–1.46)*	1.20 (1.01–1.43)*
*Chronic cardiometabolic diseases* ^*b*^			1.24 (1.02–1.49)*	1.25 (1.04–1.51)*
*Work characteristics*				
Overtime				0.97 (0.79–1.19)
Shift work				1.03 (0.75–1.40)
Night work				0.81 (0.56–1.16)
Physical labour				1.28 (1.07–1.54)**
c-statistic	0.67	0.70	0.70	0.71

*p < 0.05; **p < 0.01; ***p < 0.001; ^a^reference category: no alcohol consumption; ^b^diabetes, myocardial infarction, stroke or hypertension

All models additionally include SRH at baseline and survey as covariates, estimates are not shown

### Association of job insecurity with impaired SRH – Interaction analyses

The interaction analysis showed differences in the association between job insecurity at baseline and impaired SRH at follow-up, depending on the time-to-follow-up (p for interaction 0.035 in the fully adjusted model 4, data not shown). The relationship of job insecurity at baseline with impaired SRH at follow-up was only observed in the last survey (S3) with the shortest follow-up period (14 years on average; Table 3). For participants in S1 (time-to-follow-up 24 years on average) and those in S2 (time-to-follow-up 19 years on average), job insecurity was not significantly associated with impaired SRH in the fully adjusted model 4 (OR = 0.98, 95% CI 0.73–1.32, *p* = .89 / OR = 1.20, 95% CI 0.89–1.62, *p* = .24). However, participants from S3 who reported job insecurity at baseline had a significantly higher risk of impaired SRH 14 years later in model 4 (OR = 1.58, 95% CI 1.17–2.13, *p* = .003).

**Table 3:** Association of job insecurity with impaired SRH at follow-up, stratified for the surveys S1 (*n* = 1336), S2 (*n* = 1440) and S3 (*n* = 1580)

		Model 1 OR (95% CI)	Model 2 OR (95% CI)	Model 3 OR (95% CI)	Model 4 OR (95% CI)
*S1:*					
Job Insecurity		0.97 (0.73–1.30)	0.98 (0.73–1.32)	0.98 (0.73–1.32)	0.98 (0.73–1.32)
c-statistic		0.66	0.68	0.68	0.68
*S2*					
Job Insecurity		1.21 (0.91–1.62)	1.21 (0.90–1.63)	1.21 (0.90–1.62)	1.20 (0.89–1.62)
c-statistic		0.68	0.71	0.71	0.72
*S3*					
Job Insecurity		1.60 (1.20–2.15)**	1.60 (1.19–2.14)**	1.60 (1.19–2.16)**	1.58 (1.17–2.13)**
c-statistic		0.71	0.73	0.73	0.74

**p < 0.01; models include same covariates as in Table 2, estimates are not shown for reasons of readability of table

### Association of job insecurity with impaired SRH – Sensitivity analyses

We conducted sensitivity analyses to assess the robustness of our findings (Additional file 1: Table S1). We used job insecurity with the original three reply categories instead of the dichotomous variable in the logistic regression and estimated ORs of 1.15 (95% CI 0.96–1.37, *p* = .136) for “yes, sometimes” and 1.61 (95% CI 1.16–2.24, *p* = .005) for “yes, frequently” compared to “no” job insecurity in model 4 (model 1–3 revealed comparable estimates).

## Discussion

The present study analysed the association of job insecurity with impaired SRH in a community-dwelling working population. It showed that job insecurity was cross-sectionally associated with impaired SRH. When we prospectively analysed the long-term association of perceived job insecurity with SRH, we found that this association was still significant after a medium follow-up time of 14 years. These associations were robust to the inclusion of a number of baseline risk factors and SRH. However, the association was attenuated in subjects who were followed-up after 19 and 24 years. This is, to the best of our knowledge, the first study to have evaluated the association between job insecurity and SRH within different long-term periods.

Results from the present study overall corroborate findings from previous cross-sectional [17–22] studies, as well as prospective studies that have established the association between job insecurity and impaired SRH both in the mid-and long-term [23–26, 29]. It is evident that time plays an important role in this relationship: findings from cross-sectional [17–22] and prospective studies, with time windows between 1 and 12 years, have convincingly shown an association [26, 27, 29]. The present study is partly in line with these findings. While job insecurity was significantly associated with impaired SRH after 14 years, results of our interaction analysis showed that a longer time-to follow-up attenuated the relationship between job insecurity and impaired SRH. Although the association was not significant in the 24- (S1) and 19- (S2) year follow-up, it is noteworthy that ORs consistently increased between S1-S3. The stronger association during shorter follow-up periods potentially implies that there may be a threshold limiting the negative association of past job insecurity experiences on subsequent SRH. There has been some debate as to the role of time lags in the assessment of associations between occupational stressor and strain [28]. A possible explanation could lie in changes to the work situation over time: it is imaginable that with growing age, employees transition into better jobs with lower job insecurity. Furthermore, it is imaginable that employees develop coping mechanisms to deal with occupational stressors, thereby attenuating their negative effect on health over time [43].

Regarding the underlying mechanisms of the long-term association between job insecurity and impaired SRH, it is imaginable that employees respond to the stress of job insecurity with physiological arousal (e.g. autonomic activation) in the short term (i.e. while experiencing job insecurity), while the accumulation of these short-term responses results in adverse consequences for health in the long-term [23, 25]. Indeed, studies have found SRH to be associated with physiological markers of chronic stress and inflammation [16, 18]. Results from the present study however suggest that this potential physiological link may be limited in time, as no association between job insecurity and SRH was found beyond 14 years. Considering that the self-assessment of SRH also reflects the resources available to a person (e.g. education, social support, perceived control) [16], it is imaginable that more resources are acquired over the life-span, hence buffering the stressor-strain relationship in the long-term. The results of the sensitivity analysis might offer a further explanation (Additional file 1: Table S1), as it revealed that compared to participants without job insecurity, those who reported to “frequently” experience job insecurity, had a substantially higher and significant risk for impaired SRH at follow up than those who only “sometimes” experienced job insecurity. This indicates that the intensity or frequency of exposure might play a role in the association between job insecurity and impaired SRH, corroborating patterns from previous studies [23, 25, 30].

A further explanation for the prospective association between job insecurity and impaired SRH may lie in the socio-economic status (SES) of employees in two ways. First, those exposed to job insecurity could be more likely to have lower income and wealth in the first place and thereby may be at a higher risk for precarious employment contracts. Indeed, low SES has been identified as an antecedent for both job insecurity [7] as well as impaired SRH [15]. However, controlling for both SES and SRH at baseline in the present study may partly account for this potential causal pathway. Second, unhealthy employees may be more likely to experience job insecurity, therefore being at a higher risk of developing impaired SRH in the long-term. However, the unhealthy worker effect is not supported by the data, as there were no significant differences in terms of health behaviour, obesity or chronic cardiometabolic diseases between employees exposed vs. not exposed to job insecurity and baseline SRH was controlled for in all models. Moreover, additional adjustments for life-style choices, chronic cardiometabolic diseases and work characteristics had little effect on the prospective association between job insecurity at baseline and impaired SRH at follow-up (Table 2), strengthening the notion that job insecurity is independently negatively associated with impaired SRH in the long-term. This is in line with findings from the most recent review of longitudinal studies on job insecurity and health, where the authors conclude that there is clear evidence for normal causation (i.e. job insecurity influences subsequent health) rather than reverse causation [11].

Nevertheless, the single assessment of job insecurity in the present study does not allow for conclusions as to the length or frequency of exposure to job insecurity or potential changes to the working situation that might have occurred between baseline- and follow-up assessments. However, multiple exposure to job insecurity are likely to further strengthen the association with impaired SRH, as reported in other studies [23, 30]. While the follow-up assessment in 2009 coincided with the global economic crisis, the labour market in Germany was not as strongly affected as other countries due to the fact that social partners negotiated wage restraints in collective bargaining agreements or jobs pacts, in order to prevent job losses [44]. It is nevertheless imaginable that some participants experienced spells of unemployment between baseline and follow-up assessments, which could also account for impaired SRH at follow-up. There is evidence to suggest that employment status does not strongly alter the relationship between job insecurity and subsequent health: in a prospective study on the association between job insecurity and SRH after three and nine years of follow-up (two US samples), employment status prior to, during or at follow-up were not significant predictors in the regression analyses [23]. Virtanen et al. found in a Finnish sample that job insecurity was detrimental to health in both permanent and temporary employees [30]. In another Finnish sample, Griep et al. found that compared to secure permanent employment both insecure permanent employment and long-term unemployment were similarly detrimental to SRH [21].

In line with to current findings [7, 19], job insecurity was associated with younger age, low SES (low educational level, physical labour) and impaired SRH at baseline (Table 1). Interestingly, men reported job insecurity more often than women in the present sample. This is somewhat surprising considering that most studies to date have not found gender-specific differences in the experience of job insecurity [7]. It is imaginable that men in the present sample were more worried about keeping their jobs, as they were generally the main income providers in a family. A recent report by the German Federal Statistical Office has shown that even in 2013 men were the main income earner in 77% of couples in Germany, a decrease of 2% since 2003 [45].

A proportion of 42% of employees in the total sample reported job insecurity at baseline, similar to levels reported for Germany between 1985 and 1995 in an OECD report (38% on average) [46]. Given the high degree of labour market regulation and favourable welfare typology in Germany at the time, this may seem surprising. However, considering that job insecurity results from an individual’s subjective appraisal of a given situation and can occur in the absence of an objective trigger [4], there are a number antecedents that have been associated with job insecurity, such as self-perceived employability [47] and organizational change [7]. Moreover, since men reported job insecurity significantly more often than women in the present sample (Table 1) and missing data was significantly associated with being male, there might be an over- or underestimation of the prevalence of job insecurity.

In line with previous findings, the multivariate analyses revealed that SRH at follow-up was associated with older age, SES (low educational level, physical labour), health-behaviour (smoking, obesity, physical inactivity) chronic cardiometabolic diseases and SRH at baseline (data not shown) [35, 36, 48]. Moreover, dropouts due to missing data at baseline or follow-up were significantly older, therefore potentially more likely to be in worse health at follow-up. Considering further that impaired SRH increased with age in our study, the number of participants with impaired SRH at follow-up might be underestimated.

### Strengths & Limitations

The strengths of the present study include its population-based sample, its large sample size, a standardized, quality-controlled data collection, the control for SRH at baseline and the long follow-up periods. A further strength lies in the inclusion of a number of risk factors to job insecurity and SRH, considering that SRH is influenced by both life-style choices (e.g. physical activity, smoking) as well as objective health status (e.g. chronic cardiometabolic diseases) [48, 49].

Although the majority of research on the health-effects of job insecurity is based on single item measures of job insecurity [8], the use of a single item is a limitation of the study. While in their meta-analysis on the health effects of job insecurity, Sverke et al. did not find significant differences in terms of effects for the job insecurity-health relationship between studies that used single items, vs. multiple items, they conclude that using multiple-item measures leads to stronger associations between job insecurity and its outcomes [4]. This in turn may suggest that the associations found in the present study may be underestimated. Furthermore, the question used to assess SRH in the present study is slightly different from the widely used measure in that it refers to physical health, rather than general health. However, a number of studies have shown that SRH is highly correlated with physical health [36, 50] and that respondents also relate their overall health rating to bodily symptoms (e.g. tiredness, physical functioning) [15, 33]. A further limitation lies in the fact that other psychosocial work characteristics and structural variables pertaining to the industry of employment and the occupational position of the participants were not included in the analyses, even though they relate to job insecurity. However, controlling for SES in the analyses may partly compensate for this. Moreover, job insecurity has been established as an independent occupational stressor, over and above other psychosocial stressors at work [10, 51]. Finally, a bias due to missing information on follow-up (*n* = 2139) could not be excluded.

## Conclusion

The consideration of different follow-up timelines within a sample recruited under comparable circumstances has allowed us to explore the potential duration of the association between job insecurity and impaired SRH. We found that experiencing job insecurity during working life was independently and significantly associated with impaired SRH after 14 years, but not after 19 and 24 years. Our findings support the notion of an active management of job insecurity within the working environment, in order to preserve the health and quality of life of employees in the long-term. More longitudinal research using multiple measurement points is needed, which allows for a closer analysis of the development of both job insecurity, health and relevant covariates over time, in order to confirm these results. In addition, studies evaluating preventive approaches with regard to job insecurity within working environments are warranted. In the wake of an increasing working-life span and a rise in dynamic working conditions, it is in the interest of all stakeholders to reduce the exposure to job insecurity.

## Additional file

Additional file 1: **Table S1.** Association of job insecurity (3 reply categories, “no, never” as reference category) with diminished SRH at follow-up (*n* = 4356). (DOCX 20 kb)

## Abbreviations

MONICA/KORA: Monitoring of Trends and Determinants in Cardiovascular Disease/Cooperative Health Research in the Region of Augsburg
SRH: Self-rated health
WHO: World Health Organization
