# Supplementary material for: The impact of job insecurity on long-term self-rated health – results from the prospective population-based MONICA/KORA study
Source: BMC Public Health. 2018 Jun 18;18:754. doi: 10.1186/s12889-018-5621-4 (PMC6006929; doi:10.1186/s12889-018-5621-4)
Supplement: Supplementary file 1 — Table S1. Association of job insecurity (3 reply categories, “no, never” as reference category) with diminished SRH at follow-up (n = 4356). (DOCX 20 kb) [file 12889_2018_5621_MOESM1_ESM.docx]

Table S1: Association of job insecurity (3 reply categories, “no, never” as reference category) with diminished SRH at follow-up (n=4,356)

| Risk factor | | Model 1  OR (95% CI) | Model 2 OR (95% CI) | Model 3 OR (95% CI) | | Model 4 OR (95% CI) | |  |
| --- | --- | --- | --- | --- | --- | --- | --- | --- |
| Job Insecurity (1= “yes, frequently“)  Job Insecurity (2=“yes, sometimes“) | | 1.58 (1.13-2.19)**  1.15 (0.97-1.37) | 1.62 (1.17-2.25)**  1.16 (0.97-1.39) | 1.62 (1.16-2.25)**  1.16 (0.97-1.39) | | 1.61 (1.16-2.24)**  1.15 (0.96-1.37) | |  |
| *Socio-demographic characteristics* | |  |  |  | |  | |  |
| Age (years) |  | 1.04 (1.03-1.05)*** | 1.04 (1.03-1.05)*** | | 1.04 (1.03-1.05)*** | | 1.04 (1.02-1.05)*** | |
| Male sex | | 0.97 (0.82-1.16) | 1.02 (0.85-1.23) | 1.05 (0.88-1.26) | | 1.08 (0.89-1.31) | |  |
| Low educational level | | 1.54 (1.28-1.87)*** | 1.43 (1.18-1.73)*** | 1.42 (1.18-1.72)*** | | 1.35 (1.10-1.64)** | |  |
| *Lifestyle characteristics* | |  |  |  | |  | |  |
| Smoking | |  | 1.67 (1.39-2.01)*** | 1.67 (1.39-2.02)*** | | 1.66 (1.38-2.01)*** | |  |
| Moderate alcohol consumption^+^ | |  | 0.95 (0.76-1.19) | 0.96 (0.77-1.20) | | 0.96 (0.77-1.21) | |  |
| High alcohol consumption^+^ | |  | 1.03 (0.81-1.32) | 1.03 (0.81-1.31) | | 1.02 (0.80-1.31) | |  |
| Obesity | |  | 1.78 (1.42-2.24)*** | 1.71 (1.36-2.15)*** | | 1.67 (1.32-2.10)*** | |  |
| Physical inactivity | |  | 1.23 (1.04-1.46)* | 1.23 (1.03-1.46)* | | 1.20 (1.01-1.43)* | |  |
| *Chronic cardiometabolic diseases^#^* | |  |  | 1.23 (1.02-1.49)* | | 1.25 (1.03-1.51)* | |  |
| *Work characteristics* | |  |  |  | |  | |  |
| Overtime | |  |  |  | | 0.97 (0.79-1.19) | |  |
| Shift work | |  |  |  | | 1.02 (0.75-1.39) | |  |
| Night work | |  |  |  | | 0.80 (0.55-1.14) | |  |
| Physical labour | |  |  |  | | 1.28 (1.06-1.54)** | |  |
| c-statistic | | 0.68 | 0.71 | 0.71 | | 0.71 | |  |

***p<0.05; **p<0.01; ***p<0.001; ^+^reference category: no alcohol consumption; ^#^** **diabetes, myocardial infarction, stroke or hypertension**

**All models additionally include SRH at baseline and survey as covariates, estimates are not shown**
